# Chronic High Glucose Concentration Induces Inflammatory and Remodeling Changes in Valvular Endothelial Cells and Valvular Interstitial Cells in a Gelatin Methacrylate 3D Model of the Human Aortic Valve

**DOI:** 10.3390/polym12122786

**Published:** 2020-11-25

**Authors:** Letitia Ciortan, Razvan Daniel Macarie, Sergiu Cecoltan, Mihaela Vadana, Monica Madalina Tucureanu, Andreea Cristina Mihaila, Ionel Droc, Elena Butoi, Ileana Manduteanu

**Affiliations:** 1 Institute of Cellular Biology and Pathology “Nicolae Simionescu”, Biopathology and Therapy of Inflammation, 8, B.P. Hasdeu Street, P.O. Box 35-14, 050568 Bucharest, Romania; letitia.ciortan@icbp.ro (L.C.); razvan.macarie@icbp.ro (R.D.M.); sergiu.cecoltan@icbp.ro (S.C.); mihaela.vadana@icbp.ro (M.V.); monica.pirvulescu@icbp.ro (M.M.T.); andreea.mihaila@icbp.ro (A.C.M.); ileana.manduteanu@icbp.ro (I.M.); 2 Cardiovascular Surgery Department, Central Military Hospital, 010825 Bucharest, Romania; ionel.droc@gmail.com

**Keywords:** gelatin methacrylate, 3D model, human valvular endothelial cells, human valvular interstitial cells, high glucose levels, cytokines, cell adhesion molecules, matrix metalloproteinase, protein kinase C, calcific aortic valve disease

## Abstract

Calcific aortic valve disease (CAVD), a degenerative disease characterized by inflammation, fibrosis and calcification, is accelerated in diabetes. Hyperglycemia contributes to this process by mechanisms that still need to be uncovered. We have recently developed a 3D model of the human aortic valve based on gelatin methacrylate and revealed that high glucose (HG) induced osteogenic molecules and increased calcium deposits in a pro-osteogenic environment. To further understand the events leading to calcification in diabetic conditions in CAVD, we analyzed here the inflammatory and remodeling mechanisms induced by HG in our 3D model. We exposed valvular endothelial cells (VEC) and interstitial cells (VIC) to normal glucose (NG) or HG for 7 and 14 days, then we isolated and separated the cells by anti-CD31 immunomagnetic beads. The changes induced by HG in the 3D model were investigated by real-time polymerase chain reaction (RT-PCR), Western blot, enzyme-linked immunosorbent assay (ELISA) and immunofluorescence. Our results showed that HG induced expression of different cytokines, cell adhesion molecules and matrix metalloproteinases in VEC and VIC. In addition, protein kinase C was increased in VEC and VIC, indicating molecular mechanisms associated with HG induced inflammation and remodeling in both valvular cells. These findings may indicate new biomarkers and targets for therapy in diabetes associated with CAVD.

## 1. Introduction

Calcific aortic valve disease (CAVD) is a degenerative disease characterized by inflammation, fibrosis and calcification, leading to the hardening of the aortic valve leaflet, orifice narrowing and stenosis. Diabetes mellitus (DM) has been reported to represent a risk factor for aortic stenosis (AS) [1]. Studies that investigated the influence of DM on patients with CAVD [2] showed that it can influence the natural course and increases the progression rate of AS [3,4,5]. Moreover, the prevalence of AS and DM is high and expected to increase further in the future.

Accumulating data indicate that AS is an active process similar to atherosclerosis involving lipid infiltration, inflammation, osteoblastic transition of valve interstitial cells and active leaflet calcification [6]. The initial stage of degenerative AS has more similarities with atherosclerosis than the progressive phase, where fibrosis and calcification are more prominent. Therefore, the presence of macrophages, mast cells, CD4^+^ T cells and CD8^+^ T cells was detected in the surgically removed calcific aortic valves [7] as in atherosclerosis plaque. Moreover, the connection between lipids and the development of degenerative AS was presumed and sustained by detection of diffuse atherosclerotic lesions in the aortic leaflets of patients with familial hypercholesterolemia and no other atherosclerotic risk factors [8]. Accordingly, the contributing role of elevated LDL (low-density lipoproteins) and the risk of both vascular atherosclerosis and AS was presented in the Cardiovascular Health Study [9]. 

Despite of these similarities with atherosclerosis, the aortic valve has some properties that differ from the vascular wall [10]. Therefore, the tissue and cellular organization as well as the hemodynamic stress imposed upon the aortic valve are dissimilar from the vasculature. Another important issue is that statins, although efficient in reducing clinical events in patients with atherosclerosis, are inefficient in CAVD [11,12,13]. In diabetes, CAVD is accelerated by mechanisms that still need to be understood. Hyperglycemia, which defines the diabetic state and is common to type 1 and type 2 diabetes, may have a leading role.

The main cellular components of the aortic valve are valvular endothelial cells (VEC) and valvular interstitial cells (VIC). It has been demonstrated that high glucose is a pro-inflammatory agent for VEC, inducing VEC dysfunction. Therefore, it was shown that high glucose enhanced monocyte adhesion to the valvular endothelial cells by mechanisms involving intercellular Adhesion Molecule 1 (ICAM-1), E-selectin and CD 18 and that in high glucose conditions VEC are more adhesive to monocytes than aortic endothelial cells. These data may explain, in part, cardiac valves propensity to accelerated atherosclerosis in diabetes [14]. Other studies that investigated the effect of hyperglycemia in aortic valves found that transient hyperglycemia enhanced the synthesis of proinflammatory phospholipids and of coagulation factors, which may lead to AS in patients with poorly controlled diabetes [15,16].

Multiple VIC phenotypes have been observed in different stages of CAVD. The quiescent VIC phenotype seen in normal aortic valves is altered following injury or stress into an activated myofibroblast-like phenotype. CAVD progression leads to further alteration of this phenotype into an osteoblast-like cell, responsible for the pronounced calcification process. Previous in vitro studies showed that VIC remain quiescent in soft hydrogels and become activated on stiffer substrates and in 2D culture [17]. Moreover, studies of cellular communication of porcine VIC-VEC showed that VEC can prevent VIC calcification in osteogenic media, reducing expression of osteogenic molecules Runt-related transcription factor (Runx2) and osteocalcin (OSC), possibly due to NO signaling [18,19]. 

In this context, we developed a 3D cell culture model from gelatin methacrylate with encapsulated VIC, ensuring a quiescent VIC phenotype, and VEC seeded on the surface, that provide proper inter-cellular communication. Using this 3D model, we have recently shown that HG exposure triggered Bone morphogenetic proteins (BMP) and Transforming growth factor beta (TGF-β) signaling pathways, leading to increased expression of osteogenic molecules BMP-2/4, osteocalcin, osteopontin, SMADs and Runt-related transcription factor (RUNX), resulting in increased calcium deposits in a pro-osteogenic environment [20]. There is also evidence that inflammation and osteogenesis are synergic in early CAVD [6,15]. Therefore, using a diabetic mouse model, we have recently shown that inflammatory and osteogenic makers as well as markers of valvular cell activation are increased very early, at 4 days after diabetes onset [15]. In addition, diabetes was found to be associated with increased inflammation within AS valves, as demonstrated by increased C-reactive protein (CRP) expression, which may contribute to faster AS progression [1]. The importance of inflammation in valve calcification in diabetic conditions was also underlined by other studies which highlighted that diabetes is associated with low grade inflammation and that fundamental links exist between diabetes, inflammation, innate immune system activation and endothelial dysfunction [21]. However, the association of inflammation, diabetes and calcification was not extensively investigated in the aortic valve, consequently the mechanisms are largely unknown. Our hypothesis is that the unique modifications induced by diabetes in the inflammatory and remodeling processes in valvular cells may significantly contribute to the initiation and accelerated progression of CAVD.

The aim of the present study was to investigate inflammatory and remodeling molecules induced by HG in VEC and VIC using our previously described 3D model [20]. For this purpose, we evaluated the effect of diabetic stimuli on the gene and protein expression of different inflammatory mediators and on molecules involved in matrix remodeling. Moreover, since endothelial cells adhere to the basement membrane via integrins, a family of transmembrane heterodimeric glycoproteins that physically link the extracellular matrix (ECM) with the cell surface and the intracellular cytoskeleton, we investigated the effect of high glucose on integrins previously found to be modified in valvular disease (i.e., α4β1, αvβ3, αvβ5).

Our results showed that chronic HG exposure of 3D constructs with VEC and VIC amplify inflammation by increasing the expression of cytokines: IL-1β, TNF-α, MCP-1 and IL-8; of cell adhesion molecules (CAM): VCAM-1 and E-selectin; and of integrin: α4, αv, β1, β3 and β5 in VEC and VIC. Moreover, HG induces VIC remodeling by increasing laminin and collagen III as well as MMP-1 and MMP-13 expression in the 3D model of the human aortic valve. The phosphorylated form of protein kinase C PCK was also found increased by high glucose in VIC and VEC and involved in IL-1β overexpression. Our results may indicate new biomarkers and new targets for therapy in diabetes associated with CAVD.

## 2. Materials and Methods

### 2.1. Cells

Primary human valvular endothelial cells (VEC) were obtained from aortic valve leaflets extracted from patients undergoing aortic valve replacement surgery. Samples were collected with the consent of patients, according to the protocol of Dr. Carol Davila Central Military Emergency University Hospital and the principles regarding human sample use in experiments outlined in the Declaration of Helsinki.

Calcified regions of valve cusps were removed from samples and normal leaflet tissue was washed briefly in phosphate-buffered saline (PBS), followed by collagenase I (Merck, Darmstadt, Germany) enzymatic digestion. Cells isolated by this procedure were centrifuged and plated into 24 well plates coated with 1% gelatin from porcine skin (Sigma-Aldrich, Saint Louis, MO, USA) and cultured in EGM-2 Endothelial Cell Growth Medium (Lonza, Basel, Switzerland) supplemented with 10% fetal bovine serum (FBS) and 1% penicillin/streptomycin (Gibco, Thermo Fisher Scientific, Waltham, MA, USA) until confluent. Because the resulting cell population was a mixture of valvular cells, VEC were isolated by labeling with CD31^+^ magnetic beads (Miltenyi Biotec, Bergisch Gladbach, Germany). The resulting VEC population was cultured in EGM-2 media supplemented with 10% FBS and 1% P/S.

Human valvular interstitial cells (VIC) were purchased from Innoprot (no. P10462, Bizkaia, Spain) and cultured according to the manufacturer’s instructions. 

### 2.2. Three-Dimensional Cell Culture Model

**Figure 1:**
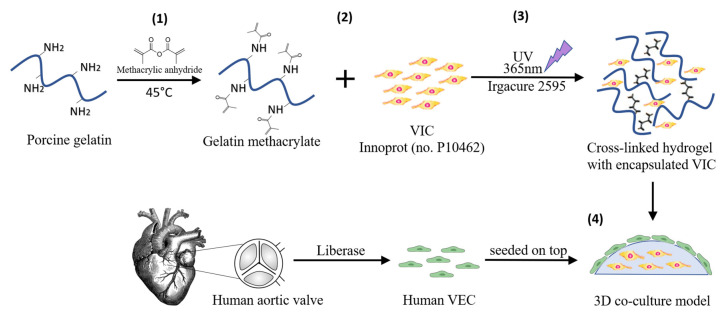
Schematic representation of 3D cell culture model preparation. (**1**) Gelatin methacrylate synthesis; (**2**) valvular interstitial cells (VIC) encapsulation in hydrogel (2 × 106 cells/mL); (**3**) Cross-linking by ultraviolet (UV) exposure; (**4**) valvular endothelial cells (VEC) seeding on top of the cross-linked hydrogel.

To better simulate the native environment and aortic valve ECM, we used a 3D cell culture model (Figure 1) obtained from methacrylate powdered type A gelatin from porcine skin (Sigma-Aldrich, Saint Louis, MO, USA), as described in [20]. 0.1% photo-initiator (Irgacure 2595, Sigma-Aldrich, Saint Louis, MO, USA) was added to the hydrogel and VIC (2 × 10^6^ cells/mL) were mixed in the hydrogel solution and cross-linked by 60 s UV exposure. The next day, VEC were seeded on each 3D construct at a density of 5 × 10^4^/cm^2^ and kept in culture until they reached confluence. Constructs were then placed in DMEM with normal (5 mM–non-diabetic condition) and high (25mM–diabetic-type condition) glucose, supplemented with 10% FBS and 1% P/S/A and maintained in culture for 7, 14 or 21 days. Cells were extracted by enzymatic digestion using Liberase (Roche, Basel, Switzerland) and total cell population was separated into VIC and VEC by human CD31^+^ magnetic beads (Miltenyi Biotec, Bergisch Gladbach, Germany).

### 2.3. Gene Expression Analysis by Real-Time Polymerase Chain Reaction (RT-PCR)

Total cellular RNA was isolated from VIC and VEC using PureLink RNA Mini Kit (Thermo Fisher Scientific, Waltham, MA, USA). First-strand cDNA synthesis was performed using 1 µg of total RNA and MMLV reverse transcriptase in accordance with the manufacturer’s protocol (Invitrogen, Carlsbad, CA, USA). Amplification of cDNA and quantification of gene expression was performed on a LightCycler 480 Real-Time Polymerase Chain Reaction (PCR) System (Roche, Basel, Switzerland), using SYBR Green I technology and specific primers (Table 1). mRNA was normalized to β-actin mRNA and relative quantification was performed using the comparative CT method. Results were expressed as arbitrary units.

### 2.4. Western Blotting

VIC and VEC cell lysates were prepared by solubilization in Pierce™ IP Lysis Buffer (Thermo Fisher Scientific, Waltham, MA, USA) according to the manufacturer’s instructions. Protein samples obtained in this way were resolved by electrophoresis on 10% polyacrylamide gels and then transferred to nitrocellulose membranes using a Trans Blot Semi-Dry system. Blots were blocked in Tris-buffered saline (TBS) with 5% bovine serum albumin (BSA, Sigma-Aldrich, Saint Louis, MO, USA) and incubated overnight with specific anti-human phospho-PKCα/β II (Thr638/641) (#9375, Cell Signaling Technology, Danvers, MA, USA) and β-actin (A5060, Sigma-Aldrich, Saint Louis, MO, USA). Blots were incubated with secondary antibodies conjugated with Horseradish Peroxidase (HRP) and developed using SuperSignal™ West Dura Extended Duration Substrate (Thermo Fisher Scientific, Waltham, MA, USA). Image acquisition was undertaken using ImageQuant LAS 4000 (GE Healthcare, Chicago, IL, USA) and optical density was measured with ImageJ software [22].

### 2.5. Enzyme-Linked Immunosorbent Assay (ELISA)

Cytokine levels were measured in conditioned media collected on day 7 and 14 from 3D constructs. Media was centrifuged at 3000 rpm for 10 min and cell debris was discarded. Supernatant aliquots were stored at −80 °C until use.

An enzyme-linked immunosorbent assay (DuoSet and Quantikine ELISA Kits, R&D Systems, Minneapolis, MN, USA) for MCP-1, IL-1β, IL-8 and TNF-α was performed according to the manufacturer’s protocol.

### 2.6. Immunofluorescence

On days 7 and 14, 3D constructs were removed from culture and fixed for 1 h in 4% paraformaldehyde in PBS. Cryoprotection was performed for each sample by successive incubation steps in phosphate buffer and 5%, 10%, 20% and 50% glycerol solutions. Before mounting in cryo-embedding media (Richard-Allan Scientific™ Neg-50™ Frozen Section Medium, Thermo Fisher Scientific, Waltham, MA, USA) samples were washed in 3% sucrose. Constructs were incubated in cryo-embedding media for 30 min at room temperature (RT) and then mounted on the holder in liquid nitrogen vapors. 7μm sections were collected onto glass slides and frozen until further use.

To perform immunofluorescence, cryosections were first brought to room temperature (RT) for 10 min and then fixed with 100% methanol (chilled at −20 °C), on ice, for 5 min. Samples were washed three times with PBS for 5 min, incubated in Sudan Black B (Sigma-Aldrich, Saint Louis, MO, USA) 1% in ethanol for 30″. Blocking was performed by incubating samples in PBS with 3% BSA for 30′ at RT, followed by incubation over night at 4 °C with primary anti-human antibodies: collagen III 1:200 dilution (PA5-27828, Invitrogen, Carlsbad, CA, USA), laminin α4 1:250 dilution (PA5-38938, Invitrogen, Carlsbad, CA, USA). The next day, samples were washed and incubated with Alexa Fluor 594 conjugated secondary antibodies at a dilution of 1:1000 for 30′ at RT in the dark. Samples stained only with secondary antibodies were used as negative controls. Samples were mounted in ProLong™ Gold mounting media (Thermo Scientific, Waltham, MA, USA), sealed and images were acquired at a 10× magnification using a fluorescence microscope (Olympus IX81, Shinjuku City, Tokyo, Japan) equipped with an XC50 camera.

## 3. Results

### 3.1. High Glucose (HG) Increases MCP-1, TNF-alpha, IL-1β and IL-8 Expression in Valvular Endothelial Cells (VEC) and Valvular Interstitial Cells (VIC) in the 3D Valve Model Based on Gelatin Methacrylate

**Figure 2:**
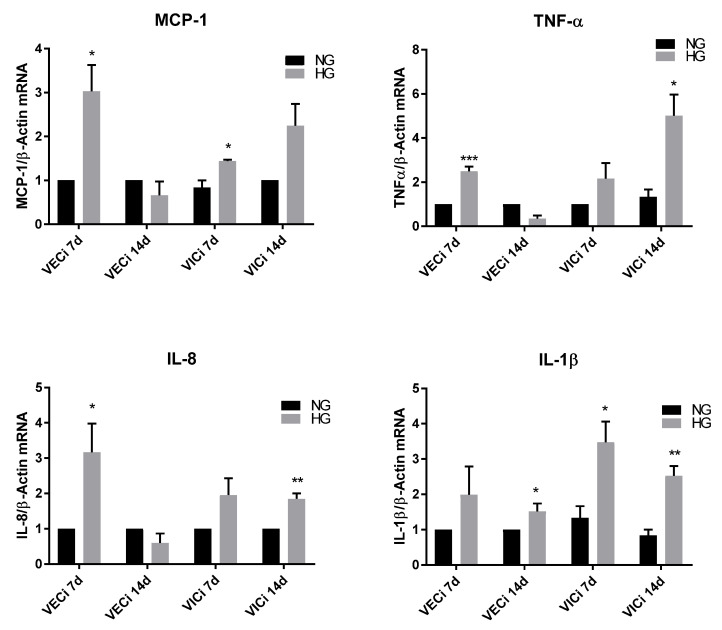
MCP-1, TNF-α, Il-8 and IL-1B gene expression in VIC and VEC isolated from 3D constructs based on gelatin, after 7 and 14 days of exposure to NG (5 mM glucose) or high glucose (HG, 5 mM glucose) conditions. The results are presented as fold change versus normal glucose (NG) condition for every type of cell and investigated time. N = 3, * *p* < 0.5, ** *p* < 0.01, *** *p* < 0.001.

The effect of HG on inflammatory cytokines expression was investigated by qPCR and ELISA, in valvular cells isolated and separated from 3D constructs after 7 and 14 days of exposure to HG or normal glucose conditions (NG). At gene expression levels, we found that after 7 days MCP-1 was significantly increased by HG both in VEC and in VIC, while at day14, MCP-1 expression was found at similar levels as in controls in VEC and at a slightly but not significantly increased level in VIC (Figure 2). In the case of TNF-α, a significant upregulation of gene expression was found in VEC after 7 days of HG exposure and in VIC after 14 days of HG exposure compared to NG (Figure 2). Similarly, the IL-8 gene expression was found to be significantly increased in VEC after 7 days of HG exposure and in VIC after 14 days, when compared to NG levels (Figure 2). Significant upregulation of IL-1β beta gene expression was detected in VIC after 7 and 14 days of HG exposure as compared to NG, and in VEC after 14 days of HG exposure (Figure 2).

**Figure 3:**
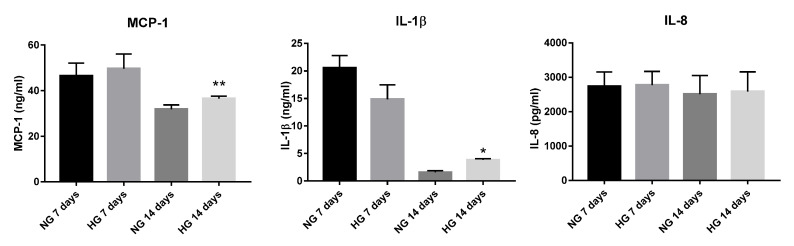
Soluble MCP-1, Il-1β and IL-8 protein secreted in the conditioned media by valvular cells from 3D-construct exposed to NG or HG conditions, as determined by enzyme-linked immunosorbent assay (ELISA). N = 3, * *p* < 0.05, ** *p* < 0.01.

The levels of cytokines released in the secretome of 3D constructs with VIC and VEC were evaluated by ELISA assay, after exposure of 3D constructs to HG/NG conditions for 7 and 14 days. The results showed that the secreted form of MCP-1 and IL-1β was significantly increased in the conditioned medium from constructs exposed 14 days to HG concentrations, compared to NG conditions (Figure 3). The IL-8 secreted levels were not significant modified in the conditioned medium from HG-exposed constructs compared with NG-exposed constructs. The secreted form of TNF-α could not be detected in our system, either in normal or in HG conditions.

### 3.2. High Glucose (HG) Increases Von Willebrand Factor (vWF) Expression in VEC and Cell Adhesion Molecules VCAM-1 and E-Selectin in both VEC and VIC

**Figure 4:**
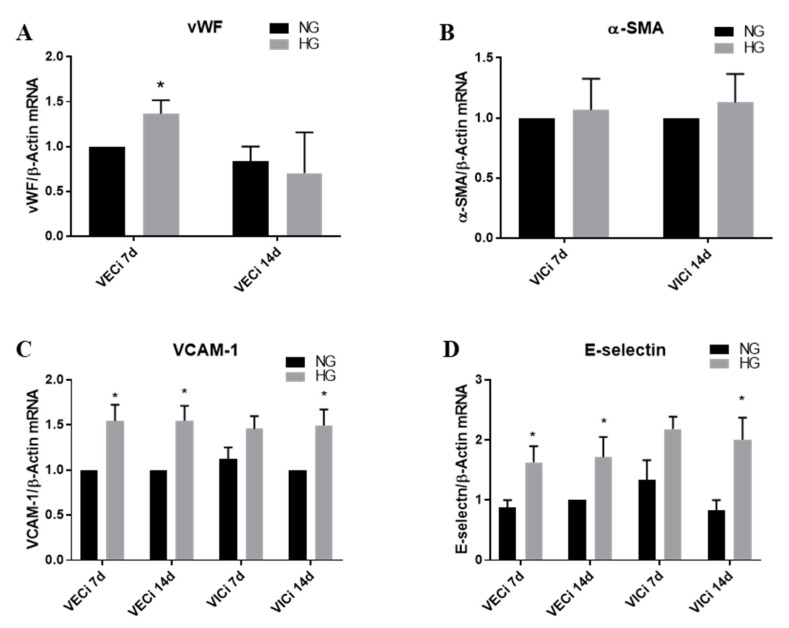
(**A**,**B**) Gene expression of von Willebrand Factor (vWF) and alpha smooth muscle actin (α-SMA) in VEC and VIC, respectively. (**C**,**D**) Gene expression of VACM-1 and E-selectin in VEC and VIC. Cells were isolated from 3D constructs after 7 and 14 days of exposure to NG (5 mM glucose) or HG (25 mM glucose) conditions. The results are presented as fold change versus NG condition for every type of cell and investigated time. N = 3, * *p* < 0.5.

In order to evaluate the endothelial and interstitial cells inflammatory status, induced by HG in our 3D valve model based on gelatin, we initially investigated the expression of endothelial cell marker–von Willebrand factor (vWF) and of activated interstitial cell marker–alpha smooth muscle actin (α-SMA). As is known, α-SMA is an accepted marker for valvular interstitial cells which switched to a myofibroblast phenotype. As shown in the Figure 4A,B, vWF gene expression was found to be increased in VEC after 7 days of HG conditions, while α-SMA gene expression was not modified in VIC after 7 or 14 days of HG exposure. Since an activated endothelium exhibits increased expression of cell adhesion molecules, we next evaluated the expression level of VCAM-1 and E-selectin in valvular cell isolated from 3D constructs exposed to NG or HG concentrations. We found that VCAM-1 expression was significantly increased in VEC from 3D constructs, both in VEC after 7 and 14 days of HG exposure and in VIC only after 14 days of HG exposure, as compared with NG (Figure 4C). E-selectin was found to be statistically significantly amplified in VEC after 7 and 14 days of HG exposure and in VIC after 14 days of HG exposure (Figure 4D).

### 3.3. High Glucose Increases α4, αV and β1 Integrin Chains Gene Expression in VEC and β3 and β5 in VIC

**Figure 5:**
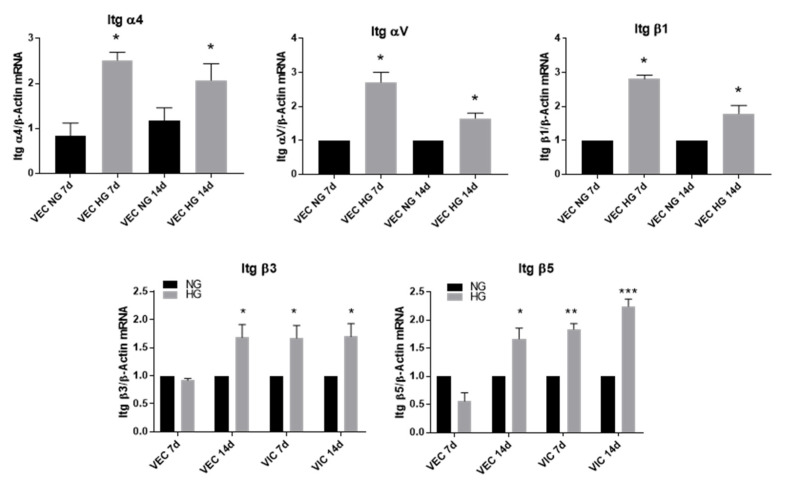
Integrin gene expression in valvular cells (VEC and VIC) in the 3D model exposed to NG or HG concentrations of glucose for 7 and 14 days. Cells were isolated from 3D constructs after 7 and 14 days and investigated by quantitative polymerase chain reaction (qPCR). The results are presented as fold change versus NG condition for every type of cell and investigated time. N = 3, * *p* < 0.5, ** *p* < 0.01, *** *p* < 0.001.

Using quantitative polymerase chain reaction (qPCR), we demonstrated that in the 3D constructs, VEC cultured for 7 or 14 days in HG conditions overexpressed the gene expression of α4, αV and β1 integrins. Therefore, in VEC, α4 gene expression was increased 2.5-fold after a 7-day exposure to HG compared to controls and 2-fold after 14 days. Integrin αV was increased 2.5-fold in VEC from 3D constructs after 7 days of HG exposure and 1.5-fold after 14 days, while β1 gene expression was increased 3-fold after 7 days and 2-fold after 14 days of exposure to HG (Figure 5).

In VIC isolated from 3D constructs after exposure to HG conditions, the gene expression of β3 and β5 subunits were significantly increased after 7 and 14 days (Figure 5).

### 3.4. Exposure of 3D Constructs with Valvular Cells to High Glucose Induces Changes in the Gene Expression of Extracellular Matrix Proteins and Metalloproteases

**Figure 6:**
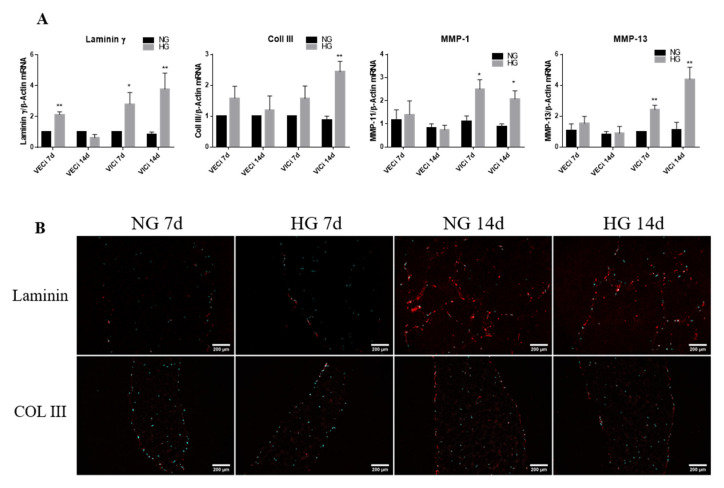
(**A**) Gene expression of extracellular matrix (ECM) proteins and MMPs (MMP-1 and MMP-13) in VEC and VIC cultured in 3D in HG versus NG glucose exposure, as evaluated by real-time PCR. The mRNA of investigated molecules was normalized to actin mRNA. The results are presented as fold change versus NG condition for every type of cell and investigated time. N = 3, * *p* < 0.5, ** *p* < 0.01. (**B**) Identification of ECM protein in 3D constructs with VEC and VIC, grown in NG or HG for 7 and 14 days. Representative images of laminin α4 chain and collagen III in sections obtained from 3D constructs grown in NG and HG for 7 and 14 days. The images were taken with an 10× objective, with filter for DAPI (nuclei–cyan) and filter Alexa 594 (laminin and collagen III–red).

To investigate the effect of HG levels on remodeling activity of valvular cells from 3D valve leaflet model, we evaluated the changes of ECM proteins: laminin γ chain, collagen I and III as well as of matrix metalloproteinases (MMP)—molecules responsible for matrix degradation. Gene expression analysis of ECM matrix proteins revealed that laminin subunit gamma was significantly increased after 7 days of HG exposure in both valvular cells VEC and VIC as compared to NG (Figure 6). Moreover, collagen III expression was significantly increased only in VIC after 14 days of exposure to HG (Figure 6), while collagen I was not found to be statistically significantly modified by HG in valvular cells (data not shown). The analysis of MMPs gene expression showed that MMP13 expression was increased in VIC exposed for 7 and 14 days to HG 2.5 and 4-fold, respectively (Figure 6A), while in VEC, its expression was not modified by 7 and 14 days of HG exposure. Similarly, the MMP-1 expression was increased only in VIC, both after 7 and 14 days of HG exposure (Figure 6A), underling that the remodeling activity is due to VIC in the 3D model. The protein expression of ECM molecules was also investigated by IF on sections obtained from 3D constructs.

The results indicate that laminin and collagen III are present in 3D constructs, with laminin observed especially on the periphery of the construct, overlapping with VEC and collagen III throughout the construct (Figure 6B). Although laminin gene expression was significantly increased by HG, the protein levels of laminin did not exhibit an increased expression in HG versus NG. These differences may be explained by the differences in laminin chains investigated by these two methods: γ chain for qPCR and α4 chain for protein. In the case of collagen III there was no differences between NG and HG both, for gene and protein expression after 7 days of HG exposure. Moreover, the matrix proteins showed increased expression in 3D constructs after 14 days compared with 7 days, suggesting increased production of these proteins over time.

### 3.5. High Glucose Activates PKC Signaling Pathway in Valvular Cells

**Figure 7:**
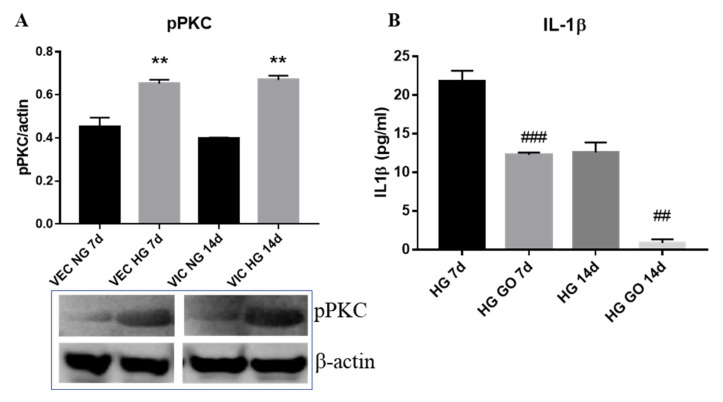
(**A**) Expression of phosphorylated forms of PKC in VEC and VIC isolated from 3D model of aortic valve. Western blot was realized on cell lysate from valvular cells isolated from 3D construct after 7 and 14 days of glucose exposure. Compared to NG (black columns), cells from HG (grey columns) exhibit increased expression of pPKC-α. N = 3, ** *p* < 0.01. (**B**) Quantification of protein levels of IL-1β released by valvular cells from the 3D model exposed to HG level in the presence or absence of PKC inhibitor, GO 6983; ## *p* < 0.01, ### *p* < 0.001.

Since our recent data showed that reactive oxygen levels were increased by HG in VIC after 7 days of exposure [20], and PCK-α is known to be activated by reactive oxygen species, we investigated whether HG affect PCK-α pathway in valvular cells. The analysis of Western Blot results (Figure 7A) showed that phosphorylated form of PKC-α was significantly increased in valvular cells from 3D constructs exposed to HG. Specifically, pPKC-α was significantly increased in VEC after 7 days of HG exposure, while in VIC the pPKC-α was significantly increased after 14 days. These data underline the fact that VEC are the first responders to higher glucose levels, followed by VIC. In order to investigate if activation of this pathway is involved in the inflammatory status of valvular cells induced by HG, we tested the effect of a potent inhibitor of PKC, GO 6983, on the IL-1β levels released in medium.

The ELISA results (Figure 7B) show that valvular cells from 3D constructs exposed to HG levels for 7 or 14 days in presence of PKC inhibitor produced low levels of IL-1β at both investigated times, as compared with IL-1β released from constructs exposed to HG without inhibitor. This result indicates that production of IL-1β is dependent on the PKC signaling pathway.

## 4. Discussion

Although is it known that CAVD is accelerated in diabetes, the early mechanisms leading to aortic valve calcification still need to be uncovered. Therefore, to understand the events accelerating CAVD in diabetic conditions, in this paper, we used the previously developed and characterized 3D model of the human aortic valve based on gelatin methacrylate [20], and analyzed the changes induced by chronic HG on the inflammatory and remodeling mediators expressed by valvular cells. The novel findings of this study are that valvular cells from the 3D model exposed to HG concentration exhibit changes of different inflammatory and remodeling molecules as follows:

1. the gene expression of cytokines (MCP-1, TNF-α, IL-8 and IL-1β), of cell adhesion molecules (VCAM-1 and E-selectin) and of integrins α4, αV, β1, β3 and β5 is significantly increased by HG in VEC after 7 days of culture; moreover, the levels of MCP-1 and IL-1β secreted in the conditioned media by valvular cells from the 3D construct are significantly increased when the constructs are exposed 14 days to HG concentrations, compared to normal glucose conditions;
2. the gene expression of cytokines (MCP-1, TNF-α, IL-8 and IL-1β), of cell adhesion molecules (VCAM-1 and E-selectin), of integrins β3 and β5 and of remodeling molecules (collagen III, laminin, MMP-1 and MMP-13) is significantly induced by HG in VIC mainly after 14 days of culture;
3. the phosphorylated form of PKC-α is elevated in both, VEC and VIC from 3D constructs exposed to HG and is involved in the production of IL-1β by valvular cells.

Previously, various studies searched for different hydrogel formulas with natural or synthetic polymers to create 3D-scaffolds for culturing VIC in 3D conditions, in order to obtain a quiescent phenotype of these cells in vitro [23]. Between all the investigated polymers used to culture VIC in 3D conditions, gelatin was one of the better choices, since VIC could proliferate and presented a quiescent phenotype [24]. Therefore, we recently developed a 3D model of aortic valve leaflet, based on gelatin methacrylate, with VIC encapsulated and VEC on top and found that VIC gained an elongated fibroblast-like morphology with a quiescent status while VEC formed an endothelial layer and stained positive for endothelial markers [20].

In this paper, the 3D model was exposed to high glucose and we started by investigating the valvular phenotype induced by HG. Interestingly, after 7 days, HG increased vWF expression in VEC, indicating VEC dysfunction, but it did not affect α-SMA in VIC even after 14 days, showing that chronic HG didn’t influence the myofibroblast phenotype of VIC. These results, are in accord with data showing that HG did not modify alpha-SMA expression in VIC exposed to HG [16,25]. However, HG induced an inflammatory phenotype of VIC by increasing progressively the expression of different cytokines and cell adhesion molecules: IL-1β, MCP-1 after 7 days; and TNF-α, IL-8, VCAM-1, E-selectin, integrin β3 and β5, after 14 days.

Regarding VEC, along with increased expression of vWF, they respond to HG faster than interstitial cells by up-regulating most of the investigated inflammatory molecules after 7 days. Therefore, MCP-1, TNF-α, IL-8, VCAM-1, E-selectin and integrins α4, αV and β1 were found to have increased in VEC after 7 days of HG exposure. After 14 days, only VCAM-1 and the integrins are still increased in VEC.

Some of the inflammatory markers found to be modified by chronic exposure of our 3D model to HG, MCP-1 and IL-1β were previously shown to be involved in CAVD. Therefore, IL-1β was found to be increased in stenotic aortic valves [26], with increased secretion induced by hyperglycemia, becoming a therapeutic target in patients with T2DM [27]. MCP-1 was shown to have important roles in CAVD and atherosclerosis [26]. In particular, MCP-1 levels were found to be substantially higher in patients with T2DM [28], and was proposed as a biomarker for aortic stenosis [29]. We extended these data, demonstrating that HG increases the gene expression and secreted form of IL-1β and MCP-1 in both VEC and VIC, in a 3D model of aortic valve leaflet, indicating that these molecules might have an important role in chronic inflammation associated with CAVD in diabetes. The mechanisms involved in the different kinetics of the inflammatory mediator’s expression and their role in CAVD progression induced by HG in our system still needs further investigation. However, our data showing an increased expression of phosphorylated PKC in valvular cells and that a specific inhibition of PKC drastically reduces IL-1β secretion from valvular cells indicate the involvement of PKC signaling pathway in HG induced inflammation. 

Concerning the effects of HG on cell adhesion molecules in our 3D construct, VCAM-1 and E-selectin were found to be increased after 7 days in VEC and after 14 days in both VEC and VIC. The integrin expression was mainly overexpressed in VEC (integrins α4, αV, β1, β3 and β5) after 7 days. In previous studies, VCAM-1, E-selectin and integrin β1, were found to have increased in human endothelial cells exposed to HG [30,31] Moreover, β1 integrin was detected in the human aortic valve leaflets [32] and in human diabetic retinal capillaries [31]. Since it was shown that in valvular cells β1 integrin is localized in focal adhesion complexes, being predominant integrin interacting with collagen fibers [33], it’s increase in HG may indicate modifications in the pattern of focal adhesion complexes, but future experiments will clarify this matter. Modified expression of β1 integrin was previously involved in valve pathology. Thus, the expression of integrin α3β1 was found increased in VEC from diseased valve [21] and calcification of VIC cultured on fibronectin and fibrin was higher when α5β1 integrin was blocked [34]. Another integrin involved in heart pathologies is αVβ3 integrin that was up-regulated in patients with myocardial infarction and was found to be associated with plaque burden in human aortic atheroma, being involved in SMC migration from media to intima, osteoclasts adhesion to the matrix and angiogenesis [35]. All these results together with our data showing that HG augmented the expression of these integrins may indicate both an increased adhesion capacity of valvular endothelium and an altered interaction of VEC with their basement membrane, underling the importance of integrin modulation in the evolution of different heart pathologies, including CAVD.

Matrix remodeling is an important process for valve calcification and we found that HG induces modification of some matrix proteins and MMPs. Therefore, while VEC exhibits changes only on laminin expression after 7 days of HG exposure, VIC expressed elevated levels of laminin and collagen III after 14 days, data demonstrating the effects of glucose on the basal lamina and extracellular matrix composition. Increased expression of MMP-1 and MMP-13 was observed only in VIC at both investigated times, suggesting an increased remodeling activity of these cells, a process involved in valve calcification. Previously, MMPs’ activation has been shown to be increased in a murine model of aortic valve disease and was associated to valvular inflammation [36]. Therefore, while MMP-1 was found to be among the most highly upregulated genes in calcific aortic valve stenosis, MMP-13 was found to be increased in pathologic conditions associated with tissue destruction, including aortic aneurism [37]. All these data may indicate that MMP-1 and 13 upregulation by HG in VIC contribute to matrix reorganization, which further lead to accelerated CAVD by mechanisms that still need to be uncovered. Since it was found that protein kinase C (PKC) regulates MMP-1 expression [38], and we found that PKC is activated in VIC from 3D constructs exposed to HG, a possible mechanism for MMPs overexpression may be dependent on PKC activation. 

Our previous study [20], indicated that, HG increased TGF beta secretion in our 3D model and activated TGF beta signaling in both VEC and VIC. Since TGF beta signaling was shown to be activated by hyperglycemia [39] and to be involved in the increase expression of cytokines and chemokines, including TNF, MCP-1, Il-8, and also ECM proteins and proteases including MMPs, we may presume that this pathway might also be involved in the cytokines and ECM remodeling molecules, revealed to be upregulated in this study. Therefore, we may presume that PCK activation and the TGF beta pathway might interplay in our system to participate in the outcome of the HG impact on valvular cells, but further experiments need to be done to clarify this matter.

In conclusion, we have shown that chronic high glucose exposure enhances the inflammatory process in valvular cells, underlying the mediators modified in valvular cells and pointing to stronger and earlier response in VEC (7 days) while greater responsiveness was noticed at a later time in VIC (14 days), which may indicate that chronic inflammation may develop and persist longer in VIC during disease progression. The inflammatory and remodeling mediators noticed may be exploited as biomarkers and targeted by future therapies in diabetes associated with CAVD.

## Figures and Tables

**Table 1:** The sequences of oligonucleotide primers used for evaluation of gene expression.

Gene	GenBank^®^ Accession Number	Sequences of Oligonucleotide Primers	Predicted Size (bp)
vWF	NM_000552	Fw: 5′-ccttgacctcggacccttatg-3′ Rv: 5′-gatgcccgttcacaccact-3′	76
VCAM-1	NM_080682	Fw: 5′-gggaagatggtcgtgatcctt-3′ Rv: 5′-ttgggcatagagaccccgtt-3′	89
TNF-α	NM_000594	Fw: 5′-ttcctcagcctcttctccttcc-3′ Rv: 5′-tgatggcagagaggaggttgac-3′	427
MCP-1	NM_002982	Fw: 5′-cagccagatgcaatcaatgcc-3′ Rv: 5′-tggaatcctgaacccacttct-3′	190
α-SMA	NM_001141945.2	Fw: 5′-actgccttggtgtgtgacaa-3′ Rv: 5′-caccatcaccccctgatgtc-3′	120
IL-1β	NM_000576	Fw: 5′-tggccctaaacagatgaagtgc-3′ Rv: 5′-tcaacacgcaggacaggtacag-3′	488
IL-8	NM_000584	Fw: 5′-actgagagtgattgagagtggac-3′ Rv: 5′-aaccctctgcacccagttttc-3′	112
E-selectin	NM_000450	Fw: 5′-agagtggagcctggtcttaca-3′ Rv: 5′-cctttgctgacaataagcactgg-3′	77
α4	NM_000885	Fw: 5′-tacagatgcaggatcggaaaga-3′ Rv: 5′-aggttctccattagggctacc-3′	75
αV	NM_002210	Fw: 5′-gctgtcggagatttcaatggt-3′ Rv: 5′-tctgctcgccagtaaaattgt-3′	136
β1	NM_002211	Fw: 5′-gtaaccaaccgtagcaaagga-3′ Rv: 5′-tcccctgatcttaatcgcaaaac-3′	98
β3	NM_000212	Fw: 5′-catgaaggatgatctgtggagc-3′ Rv: 5′-aatccgcaggttactggtgag-3′	85
β5	NM_002213.5	Fw: 5′-ggaagttcggaaacagagggt-3′ Rv: 5′-ctttcgccagccaatcttctc-3′	106
Coll III	NM_000090.3	Fw: 5′-aggtcctgcgggtaacact-3′ Rv: 5′-actttcacccttgacaccctg-3′	226
Laminin	NM_005559.4	Fw: 5′-gtgatggcaacagcgcaaa-3′ Rv: 5′-gacccagtgatattctctccca-3′	116
MMP-1	NM_002421.3	Fw:5′-aaaattacacgccagatttgcc-3′ Rv: 5′-ggtgtgacattactccagagttg-3′	82
MMP-13	NM_002427.3	Fw: 5′-actgagaggctccgagaaatg-3′ Rv: 5′-gaaccccgcatcttggctt-3′	103
β-actin	NM_001101.4	Fw: 5′-catgtacgttgctatccaggc-3′ Rv: 5′-ctccttaatgtcacgcacgat-3′	250

## References

[1] Natorska J., Wypasek E., Grudzień G., Sobczyk D., Marek G., Filip G., Sadowski J., Undas A. (2012). Does Diabetes Accelerate the Progression of Aortic Stenosis through Enhanced Inflammatory Response within Aortic valves?. Inflammation.

[2] Banovic M., Athithan L., McCann G.P. (2019). Aortic stenosis and diabetes mellitus: An ominous combination. Diabetes Vasc. Dis. Res..

[3] Kamalesh M., Ng C., El Masry H., Eckert G., Sawada S. (2009). Does diabetes accelerate progression of calcific aortic stenosis?. Eur. J. Echocardiogr..

[4] The British Diabetic Association Diabetes in the UK 2013: Key Statistics on Diabetes. https://www.diabetes.org.uk/professionals/position-statements-reports/statistics/diabetes-in-the-uk-2013-key-statistics-on-diabetes.

[5] Larsson S.C., Wallin A., Håkansson N., Stackelberg O., Bäck M., Wolk A. (2018). Type 1 and type 2 diabetes mellitus and incidence of seven cardiovascular diseases. Int. J. Cardiol..

[6] Dweck M.R., Boon N.A., Newby D.E. (2012). Calcific Aortic Stenosis: A Disease of the Valve and the Myocardium. J. Am. Coll. Cardiol..

[7] Mathieu P., Bouchareb R., Boulanger M.-C. (2015). Innate and Adaptive Immunity in Calcific Aortic Valve Disease. J. Immunol. Res..

[8] Coté N., Mahmut A., Bosse Y., Couture C., Pagé S., Trahan S., Boulanger M.C., Fournier D., Pibarot P., Mathieu P. (2013). Inflammation is associated with the remodeling of calcific aortic valve disease. Inflammation.

[9] Stewart B.F., Siscovick D., Lind B.K., Gardin J.M., Gottdiener J.S., Smith V.E., Kitzman D.W., Otto C.M. (1997). Clinical factors associated with calcific aortic valve disease. Cardiovascular Health Study. J. Am. Coll. Cardiol..

[10] Rajamannan N.M., Evans F.J., Aikawa E., Grande-Allen K.J., Demer L.L., Heistad D.D., Simmons C.A., Masters K.S., Mathieu P., O’Brien K.D. (2011). Calcific aortic valve disease: Not simply a degenerative process: A review and agenda for research from the National Heart and Lung and Blood Institute Aortic Stenosis Working Group. Executive summary: Calcific aortic valve disease-2011 update. Circulation.

[11] Cowell S.J., Newby D.E., Prescott R.J., Bloomfield P., Reid J., Northridge D.B., Boon N.A. (2005). Scottish Aortic Stenosis and Lipid Lowering Trial, Impact on Regression (SALTIRE) Investigators. A randomized trial of intensive lipid-lowering therapy in calcific aortic stenosis. N. Engl. J. Med..

[12] Rossebø A.B., Pedersen T.R., Boman K., Brudi P., Chambers J.B., Egstrup K., Gerdts E., Gohlke-Bärwolf C., Holme I., Kesäniemi Y.A. (2008). Intensive Lipid Lowering with Simvastatin and Ezetimibe in Aortic Stenosis. N. Engl. J. Med..

[13] Chan K.L., Teo K., Dumesnil J.G., Ni A., Tam J. (2010). Effect of Lipid Lowering with Rosuvastatin on Progression of Aortic Stenosis. Circulation.

[14] Simionescu N., Vasile E., Lupu F., Popescu G., Simionescu M. (1986). Prelesional events in atherogenesis. Accumulation of extracellular cholesterol-rich liposomes in the arterial intima and cardiac valves of the hyperlipidemic rabbit. Am. J. Pathol..

[15] Tucureanu M.M., Filippi A., Alexandru N., Ana Constantinescu C., Ciortan L., Macarie R., Vadana M., Voicu G., Frunza S., Nistor D. (2019). Diabetes-induced early molecular and functional changes in aortic heart valves in a murine model of atherosclerosis. Diabetes Vasc. Dis. Res..

[16] Selig J.I., Ouwens D.M., Raschke S., Thoresen G.H., Fischer J.W., Lichtenberg A., Akhyari P., Barth M. (2019). Impact of hyperinsulinemia and hyperglycemia on valvular interstitial cells—A link between aortic heart valve degeneration and type 2 diabetes. Biochim. Biophys. Acta Mol. Basis Dis..

[17] Wang H., Tibbitt M.W., Langer S.J., Leinwand L.A., Anseth K.S. (2013). Hydrogels preserve native phenotypes of valvular fibroblasts through an elasticity-regulated PI3K/AKT pathway. Proc. Natl. Acad. Sci. USA.

[18] Kennedy J.A., Hua X., Mishra K., Murphy G.A., Rosenkranz A.C., Horowitz J.D. (2009). Inhibition of calcifying nodule formation in cultured porcine aortic valve cells by nitric oxide donors. Eur. J. Pharmacol..

[19] Richards J., El-Hamamsy I., Chen S., Sarang Z., Sarathchandra P., Yacoub M.H., Chester A.H., Butcher J.T. (2013). Side-specific endothelial-dependent regulation of aortic valve calcification: Interplay of hemodynamics and nitric oxide signaling. Am. J. Pathol..

[20] Vadana M., Cecoltan S., Ciortan L., Macarie R.D., Tucureanu M.M., Mihaila A.C., Droc I., Butoi E., Manduteanu I. (2020). Molecular mechanisms involved in high glucose-induced valve calcification in a 3D valve model with human valvular cells. J. Cell. Mol. Med..

[21] Garcia C., Feve B., Ferré P., Halimi S., Baizri H., Bordier L., Guiu G., Dupuy O., Bauduceau B., Mayaudon H. (2010). Diabetes and inflammation: Fundamental aspects and clinical implications. Diabetes Metab..

[22] Schneider C.A., Rasband W.S., Eliceiri K.W. (2012). NIH Image to ImageJ: 25 years of image analysis. Nat. Methods.

[23] Zhang X., Xu B., Puperi D.S., Wu Y., West J.L., Grande-Allen K.J. (2015). Application of hydrogels in heart valve tissue engineering. J. Long Term Eff. Med. Implants.

[24] Duan B., Hockaday L.A., Kang K.H., Butcher J.T. (2013). 3D bioprinting of heterogeneous aortic valve conduits with alginate/gelatin hydrogels. J. Biomed. Mater. Res. A.

[25] Voicu G., Rebleanu D., Constantinescu C.A., Fuior E.V., Ciortan L., Droc I., Uritu C.M., Pinteala M., Manduteanu I., Simionescu M. (2020). Nano-Polyplexes Mediated Transfection of Runx2-shRNA Mitigates the Osteodifferentiation of Human Valvular Interstitial Cells. Pharmaceutics.

[26] Alushi B., Curini L., Christopher M.R., Grubitzch H., Landmesser U., Amedei A., Lauten A. (2020). Calcific Aortic Valve Disease-Natural History and Future Therapeutic Strategies. Front. Pharmacol..

[27] Larsen C.M., Faulenbach M., Vaag A., Vølund A., Ehses J.A., Seifert B., Mandrup-Poulsen T., Donath M.Y. (2007). Interleukin-1-receptor antagonist in type 2 diabetes mellitus. N. Engl. J. Med..

[28] Waheed H.J., Khalil M., Fawzi S. (2015). Evaluation of Monocyte Chemoattractant Protein-1 (MCP-1) in Type 2 Diabetes Mellitus. Int. J. Sci. Eng. Res..

[29] Kapelouzou A., Tsourelis L., Kaklamanis L., Degiannis D., Kogerakis N., Cokkinos D.V. (2015). Serum and tissue biomarkers in aortic stenosis. Glob. Cardiol. Sci. Pract..

[30] Manduteanu I., Voinea M., Serban G., Simionescu M. (1999). High glucose induces enhanced monocyte adhesion to valvular endothelial cells via a mechanism involving ICAM-1, VCAM-1 and CD18. Endothelium.

[31] Roth T., Podestá F., Stepp M.A., Boeri D., Lorenzi M. (1993). Integrin overexpression induced by high glucose and by human diabetes: Potential pathway to cell dysfunction in diabetic microangiopathy. Proc. Natl. Acad. Sci. USA.

[32] Latif N., Sarathchandra P., Taylor P.M., Antoniw J., Yacoub M.H. (2005). Localization and pattern of expression of extracellular matrix components in human heart valves. J. Heart Valve Dis..

[33] Butcher J.T., Nerem R.M. (2004). Porcine aortic valve interstitial cells in three-dimensional culture: Comparison of phenotype with aortic smooth muscle cells. J. Heart Valve Dis..

[34] Gu X., Masters K.S. (2010). Regulation of valvular interstitial cell calcification by adhesive peptide sequences. J. Biomed. Mater. Res. Part A.

[35] Jenkins W.S., Vesey A.T., Stirrat C., Connell M., Lucatelli C., Neale A., Moles C., Vickers A., Fletcher A., Pawade T. (2017). Cardiac α(V)β(3) integrin expression following acute myocardial infarction in humans. Heart.

[36] Bossé Y., Miqdad A., Fournier D., Pépin A., Pibarot P., Mathieu P. (2009). Refining molecular pathways leading to calcific aortic valve stenosis by studying gene expression profile of normal and calcified stenotic human aortic valves. Circ. Cardiovasc. Genet..

[37] Ikonomidis J.S., Jones J.A., Barbour J.R., Stroud R.E., Clark L.L., Kaplan B.S., Zeeshan A., Bavaria J.E., Gorman J.H., Spinale F.G. (2007). Expression of matrix metalloproteinases and endogenous inhibitors within ascending aortic aneurysms of patients with bicuspid or tricuspid aortic valves. J. Thorac. Cardiovasc. Surg..

[38] Sokolova O., Vieth M., Naumann M. (2013). Protein kinase C isozymes regulate matrix metalloproteinase-1 expression and cell invasion in *Helicobacter pylori* infection. Gut.

[39] Zhao L., Zou Y., Liu F. (2020). Transforming Growth Factor-Beta1 in Diabetic Kidney Disease. Front. Cell Dev. Biol..

